# Crystal structure of 2-(4-chloro­phen­yl)-2-oxoethyl 3-bromo­benzoate

**DOI:** 10.1107/S1600536814021643

**Published:** 2014-10-08

**Authors:** Imtiaz Khan, Aliya Ibrar, Shahid Hameed, Jonathan M. White, Jim Simpson

**Affiliations:** a Department of Chemistry, Quaid-i-Azam University, Islamabad 45320, Pakistan; b School of Chemistry, University of Nottingham, University Park, Nottingham NG7 2RD, England; c School of Chemistry and Bio-21 Institute, University of Melbourne, Parkville, Victoria 3052, Australia; d Department of Chemistry, University of Otago, PO Box 56, Dunedin, New Zealand

**Keywords:** crystal structure, 2-(4-chloro­phen­yl)-2-oxoethyl 3-bromo­benzoate, synthesis, π–π inter­actions, inversion dimers

## Abstract

2-(4-Chloro­phen­yl)-2-oxoethyl 3-bromo­benzoate, C_15_H_10_BrClO_3_, was synthesized in a single-step reaction by condensation of 3-bromo­benzoic acid with 2-bromo-1-(4-chloro­phen­yl)ethanone in di­methyl­formamide in the presence of tri­ethyl­amine as a catalyst. The structure consists of an aryl ketone moiety linked to an aryl ester unit by a methyl­ene group. Both units are reasonably planar (r.m.s. deviations of 0.119 and 0.010 Å for the aryl ketone and aryl ester units, respectively) and are almost orthogonal, with an angle of 88.60 (3)° between them. In the crystal, mol­ecules form five separate sets of inversion dimers. Three of these are generated by two C—H⋯O inter­actions and a C—H⋯Br contact, and form chains along *c* and along the *ab* cell diagonal. In addition, two inversion-related π–π stacking inter­actions between like aryl rings again form chains of mol­ecules but in this instance along the *bc* diagonal. These contacts generate infinite layers of mol­ecules parallel to (011) and stack the mol­ecules along the *a*-axis direction.

## Abstract 2

Packing in the title keto ester compound is dominated by the formation of inversion dimers by both non-classical hydrogen bonds and offset π–π stacking inter­actions.

## Chemical context   

Keto esters, an important class of versatile inter­mediates, have been reported to show anti­tumor activity against Ehrlich cells and HeLa cells (Kinoshita & Umezawa, 1960 ▶). They also regulate the flowering times of some plants (Kai *et al.*, 2007 ▶). Recent studies have revealed that they also exhibit inhibitory activity against two isozymes of 11β-hy­droxy­steroid de­hydro­genases (11β-HSD1 and 11β-HSD2), which catalyse the inter­conversion of active cortisol and inactive cortisone (Zhang *et al.*, 2009 ▶). Dicarbonyl compounds and their deriv­atives are also among the most versatile and frequently employed synthons in organic synthesis, especially in heterocyclic chemistry (Stanovnik & Svete, 2004 ▶; Sheibani *et al.*, 2006*a* ▶,*b* ▶, 2007 ▶; Pal *et al.*, 2008 ▶). In this work, we report the synthesis of 2-(4-chloro­phen­yl)-2-oxoethyl 3-bromo­benzoate, (1), which may be used as an effective synthon in organic chemistry.
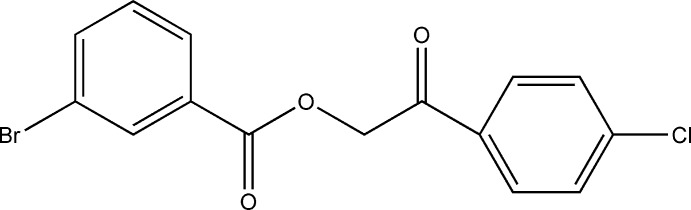


## Structural commentary   

The structure of (1) consists of an aryl ketone moiety linked to an aryl ester unit by the C8 methyl­ene group and both groupings are reasonably planar. There is an r.m.s. deviation of 0.119 Å from the best-fit plane through atoms Br1, C1–C8, O1, O2 [maximum deviation 0.2477 (11) Å for O1] while the plane of the carboxyl­ate unit subtends an angle of 15.5 (2)° to that of the bromo­benzene ring. In addition, the plane of the aryl ketone unit C8–C15, O3, Cl1 has an r.m.s. deviation of 0.010 Å [maximum deviation 0.0171 (15) Å for C15]. The aryl ketone and aryl ester planes are almost orthogonal with an angle of 88.61 (3)° between them. Bond lengths and angles in the mol­ecule are normal and are generally similar to those found in closely related mol­ecules (see for example Fun *et al.*, 2011*a* ▶; Chidan Kumar *et al.*, 2014*c* ▶).

## Supra­molecular features   

**Figure 2:**
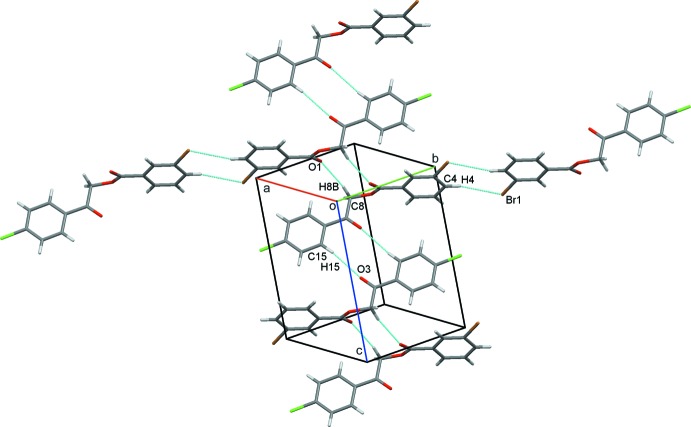
Chains of linked inversion dimers generated by C—H⋯O and C—H⋯Br hydrogen bonds, drawn as dashed lines.

**Figure 3:**
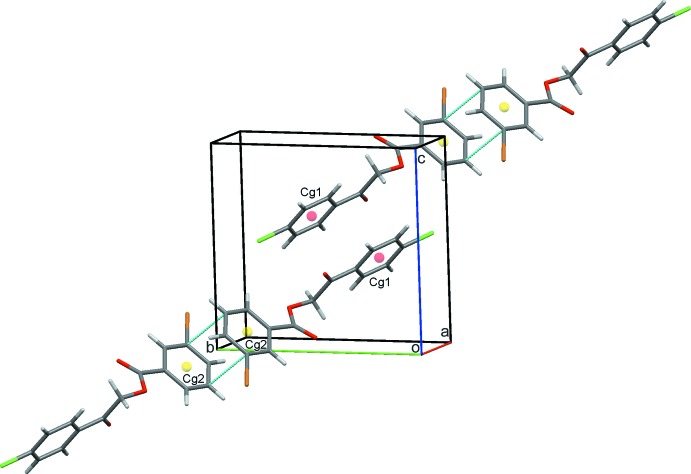
A chain of inversion dimers generated by π–π contacts, dotted green lines, between 3-bromo­phenyl and 4-chloro­phenyl rings. Ring centroids are displayed as coloured spheres.

**Figure 4:**
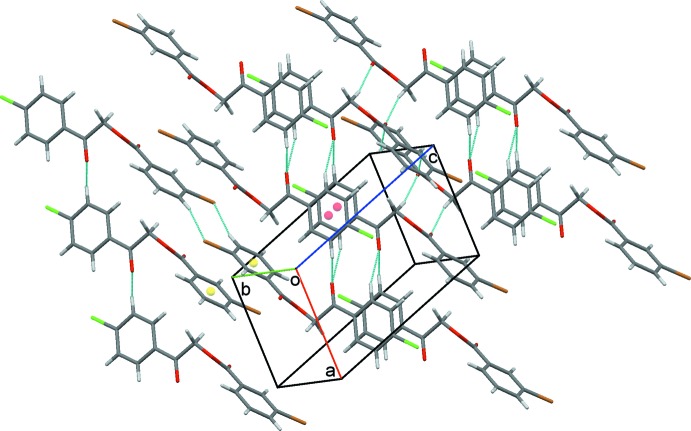
Overall packing of (1) viewed at right angles to (011).

**Figure 5:**
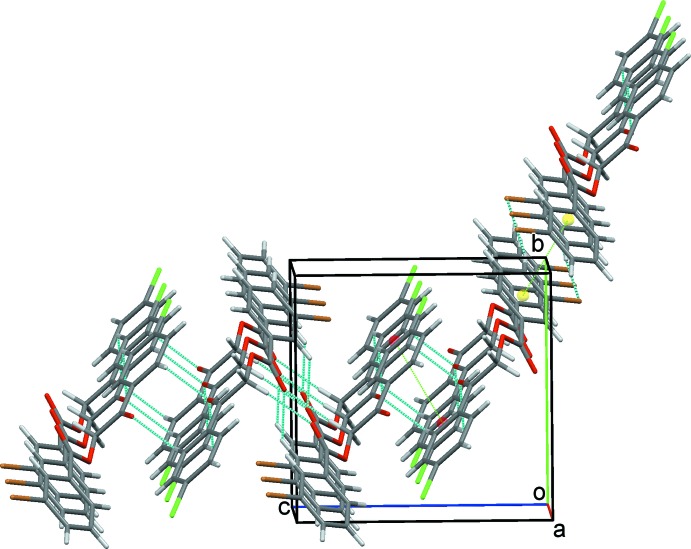
Overall packing of (1) viewed along the *a*-axis direction.

In the crystal structure, each mol­ecule forms five separate inversion dimers. C8—H8*B*⋯O1 and C15—H15 O3 hydrogen bonds each generate 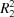
(10) rings, forming zigzag chains along *c*. Additional C4—H4⋯Br1 contacts also form inversion dimers with 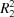
(8) rings and these combine with the C8—H8*B*⋯O1 contacts to link alternating pairs of dimers into infinite chains approximately along the *ab* cell diagonal, Table 1 ▶, Fig. 2 ▶. Inter­estingly, infinite chains of alternating inversion dimers also result from a pair of π–π stacking inter­actions between adjacent 3-bromo­phenyl rings, *Cg*1⋯*Cg*1^iv^ = 3.6987 (10) Å, and neighbouring 4-chloro­phenyl rings *Cg*2⋯*Cg*2^v^ = 3.8585 (11) Å, in this case along the *bc* diagonal, Fig. 3 ▶ [*Cg*1 and *Cg*2 are the centroids of the C1–C6 and C10–C15 rings, respectively; symmetry codes (iv) −*x*, 2 − *y*, −*z*; (v) 2 − *x*, 1 − *y*, 1 − *z*]. These contacts combine to generate extended layers of mol­ecules parallel to (011), Fig. 4 ▶, and to stack mol­ecules along the *a*-axis direction, Fig. 5 ▶.

## Database survey   

A search of the Cambridge Crystallographic Database (Groom & Allen, 2014 ▶) reveals only eight structures with the 2-oxo-2-phenyl­ethyl benzoate skeleton. These include the archetypal 2-oxo-2-phenyl­ethyl benzoate (Fun *et al.*, 2011*a* ▶), three additional 2-(4-chloro­phen­yl)-2-oxoethyl derivatives (Fun *et al.*, 2011**b* ▶;* Chidan Kumar *et al.*, 2014*a* ▶,*b* ▶) and the corresponding compound 2-(4-bromo­phen­yl)-2-oxoethyl 3-chloro­benzoate with the chloro- and bromo-substituents reversed (Chidan Kumar *et al.*, 2014*c* ▶). Interestingly, inversion-dimer formation is a feature of the packing in several of these structures.

## Synthesis and crystallization   

The preparation followed a procedure developed for the preparation of a related compound (Khan *et al.*, 2012 ▶). Tri­ethyl­amine (4–5 drops) was added at room temperature to a stirred solution of 3-bromo­benzoic acid (1.0 mmol) in *N*,*N*-di­methyl­formamide (DMF), followed by a solution of 2-bromo-1-(4-chloro­phen­yl)ethanone (1.0 mmol). The reaction mixture was stirred for 2 h. Progress of the reaction was monitored by TLC. After completion, the mixture was poured into water and the precipitated solid was filtered, dried and recrystallized (EtOAc/hexa­ne) to afford 2-(4-chloro­phen­yl)-2-oxoethyl 3-bromo­benzoate (1). The formation of keto ester (3) was indicated by the appearance of two typical stretching vibrations ν(C=O) ester (1724) and ν(C=O) keto (1698) cm^−1^, respectively and the disappearance of characteristic IR stretching absorptions ascribable to the carb­oxy­lic acid group in the region of 3400–2400 cm^−1^. In the ^1^H NMR spectrum, the signals for the aromatic protons appeared in their respective regions and the disappearance of a characteristic signal for the COOH proton confirmed the formation of the title compound (1). The ^13^C NMR spectrum displayed two characteristic signals for the keto and ester carbonyl carbon atoms at 190.7 and 165.3 p.p.m., respectively. Yield: 88%; m.p. 372–373 K; *R*_f_: 0.72 (10% EtOAc/hexa­ne); IR (ATR, cm^−1^): 3089 (C*sp* ^2^-H), 2933, 2856 (C*sp* ^3^-H), 1724 (C=O ester), 1698 (C=O ketone), 1585, 1479 (C=C Ar), 1225 (C—O); ^1^H NMR (300 MHz, CDCl_3_): δ 8.06–8.03 (*m*, 1H, Ar-H), 7.94–7.90 (*m*, 2H, Ar-H), 7.73–7.70 (*m*, 1H, Ar-H), 7.52–7.48 (*m*, 2H, Ar-H), 7.46–7.36 (*m*, 2H, Ar-H), 5.57 (*s*, 2H, CH_2_); ^13^C NMR (75 MHz, CDCl_3_): δ 190.7, 165.3, 140.6, 134.5, 133.1, 132.4, 132.0, 131.0, 129.3, 129.3, 127.3, 122.1, 66.5.

## Refinement   

All H atoms were refined using a riding model, with C—H = 0.95 Å and *U*_iso_(H) = 1.2*U*_eq_(C) for aromatic, and C—H = 0.99 Å and *U*_iso_(H) = 1.2*U*_eq_(C) for the methyl­ene H atoms.

## Supplementary Material

Crystal structure: contains datablock(s) global, 1. DOI: 10.1107/S1600536814021643/hg5410sup1.cif

Structure factors: contains datablock(s) 1. DOI: 10.1107/S1600536814021643/hg54101sup2.hkl

CCDC reference: 864789

Additional supporting information:  crystallographic information; 3D view; checkCIF report

## Figures and Tables

**Figure 1:**
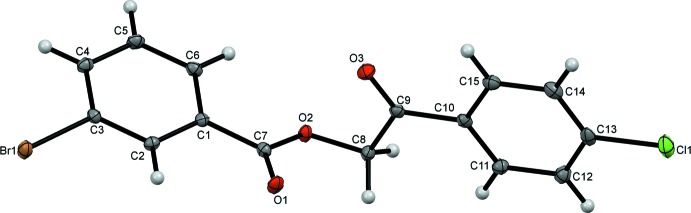
Fig, 1. The structure of (1) with displacement ellipsoids drawn at the 50% probability level.

**Table 1:** Hydrogen-bond geometry (, )

*D*H*A*	*D*H	H*A*	*D* *A*	*D*H*A*
C4H4Br1^i^	0.95	2.97	3.8762(18)	160
C8H8*B*O1^ii^	0.99	2.42	3.396(2)	168
C15H15O3^iii^	0.95	2.60	3.418(2)	144

Symmetry codes: (i) 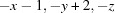; (ii) 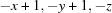; (iii) 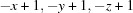.

**Table 2:** Experimental details

Crystal data	
Chemical formula	C_15_H_10_BrClO_3_
*M*_r_	353.59
Crystal system, space group	Triclinic, *P* 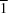
Temperature (K)	130
*a*, *b*, *c* ()	6.6797(3), 10.0238(4), 10.7851(5)
, , ()	90.980(4), 107.573(4), 92.138(3)
*V* (^3^)	687.64(5)
*Z*	2
Radiation type	Mo *K*
(mm^1^)	3.19
Crystal size (mm)	0.50 0.40 0.20

Data collection	
Diffractometer	Agilent SuperNova (Dual, Cu at zero, Atlas CCD)
Absorption correction	Multi-scan (*CrysAlis PRO*; Agilent, 2011 ▶)
*T*_min_, *T*_max_	0.505, 1.000
No. of measured, independent and observed [*I* > 2(*I*)] reflections	10822, 3327, 3062
*R*_int_	0.033
(sin /)_max_ (^1^)	0.687

Refinement	
*R*[*F* ^2^ > 2(*F* ^2^)], *wR*(*F* ^2^), *S*	0.027, 0.064, 1.05
No. of reflections	3327
No. of parameters	181
H-atom treatment	H-atom parameters constrained
_max_, _min_ (e ^3^)	0.35, 0.60

Computer programs: *CrysAlis PRO*, (Agilent, 2011 ▶), *SHELXS97* and *SHELXL2014* (Sheldrick, 2008 ▶), *Mercury* (Macrae *et al.*, 2008 ▶), *enCIFer* (Allen *et al.*, 2004 ▶), *PLATON* (Spek, 2009 ▶) and *publCIF* (Westrip, 2010 ▶).

## supplementary crystallographic information

### Crystal data

**Table:**

C_15_H_10_BrClO_3_	*Z* = 2
*M**_r_* = 353.59	*F*(000) = 352
Triclinic, *P*1	*D*_x_ = 1.708 Mg m^−^^3^
*a* = 6.6797 (3) Å	Mo *K*α radiation, λ = 0.71073 Å
*b* = 10.0238 (4) Å	Cell parameters from 6648 reflections
*c* = 10.7851 (5) Å	θ = 3.2–29.2°
α = 90.980 (4)°	µ = 3.19 mm^−^^1^
β = 107.573 (4)°	*T* = 130 K
γ = 92.138 (3)°	Block, colourless
*V* = 687.64 (5) Å^3^	0.50 × 0.40 × 0.20 mm

### Data collection

**Table:**

Agilent SuperNova (Dual, Cu at zero, Atlas CCD) diffractometer	3327 independent reflections
Radiation source: SuperNova (Mo) X-ray Source	3062 reflections with *I* > 2σ(*I*)
Mirror monochromator	*R*_int_ = 0.033
ω scans	θ_max_ = 29.2°, θ_min_ = 3.2°
Absorption correction: multi-scan (*CrysAlis PRO*; Agilent, 2011)	*h* = −8→8
*T*_min_ = 0.505, *T*_max_ = 1.000	*k* = −13→13
10822 measured reflections	*l* = −14→14

### Refinement

**Table:**

Refinement on *F*^2^	0 restraints
Least-squares matrix: full	Hydrogen site location: inferred from neighbouring sites
*R*[*F*^2^ > 2σ(*F*^2^)] = 0.027	H-atom parameters constrained
*wR*(*F*^2^) = 0.064	*w* = 1/[σ^2^(*F*_o_^2^) + (0.0307*P*)^2^ + 0.1071*P*] where *P* = (*F*_o_^2^ + 2*F*_c_^2^)/3
*S* = 1.05	(Δ/σ)_max_ = 0.001
3327 reflections	Δρ_max_ = 0.35 e Å^−^^3^
181 parameters	Δρ_min_ = −0.60 e Å^−^^3^

### Special details

**Table:**

Experimental. Absorption correction: CrysAlisPro, Agilent (2011), Empirical absorption correction using spherical harmonics, implemented in SCALE3 ABSPACK scaling algorithm.
Geometry. All e.s.d.'s (except the e.s.d. in the dihedral angle between two l.s. planes) are estimated using the full covariance matrix. The cell e.s.d.'s are taken into account individually in the estimation of e.s.d.'s in distances, angles and torsion angles; correlations between e.s.d.'s in cell parameters are only used when they are defined by crystal symmetry. An approximate (isotropic) treatment of cell e.s.d.'s is used for estimating e.s.d.'s involving l.s. planes.

### Fractional atomic coordinates and isotropic or equivalent isotropic displacement parameters (Å^2^)

**Table:**

	*x*	*y*	*z*	*U*_iso_*/*U*_eq_	
C1	0.1452 (3)	0.76183 (16)	0.09834 (16)	0.0153 (3)	
C2	−0.0463 (3)	0.73719 (17)	0.00240 (16)	0.0165 (3)	
H2	−0.0671	0.6625	−0.0562	0.020*	
C3	−0.2052 (3)	0.82374 (17)	−0.00564 (16)	0.0164 (3)	
C4	−0.1816 (3)	0.93075 (18)	0.08011 (17)	0.0200 (4)	
H4	−0.2944	0.9879	0.0739	0.024*	
C5	0.0097 (3)	0.95371 (18)	0.17577 (17)	0.0213 (4)	
H5	0.0280	1.0271	0.2356	0.026*	
C6	0.1742 (3)	0.87044 (17)	0.18485 (17)	0.0189 (3)	
H6	0.3057	0.8874	0.2496	0.023*	
C7	0.3120 (3)	0.66531 (17)	0.10555 (16)	0.0169 (3)	
C8	0.6639 (3)	0.62000 (17)	0.20718 (17)	0.0182 (3)	
H8A	0.8031	0.6678	0.2388	0.022*	
H8B	0.6573	0.5687	0.1266	0.022*	
C9	0.6368 (3)	0.52539 (17)	0.30950 (16)	0.0176 (3)	
C10	0.8001 (3)	0.42573 (17)	0.35889 (16)	0.0166 (3)	
C11	0.9739 (3)	0.41710 (18)	0.31370 (17)	0.0201 (4)	
H11	0.9919	0.4770	0.2502	0.024*	
C12	1.1213 (3)	0.32135 (19)	0.36109 (18)	0.0231 (4)	
H12	1.2400	0.3152	0.3305	0.028*	
C13	1.0919 (3)	0.23586 (18)	0.45295 (18)	0.0229 (4)	
C14	0.9204 (3)	0.24247 (19)	0.49972 (18)	0.0242 (4)	
H14	0.9029	0.1821	0.5630	0.029*	
C15	0.7757 (3)	0.33824 (19)	0.45280 (17)	0.0215 (4)	
H15	0.6584	0.3446	0.4848	0.026*	
O1	0.28187 (18)	0.55756 (12)	0.05094 (12)	0.0222 (3)	
O2	0.50195 (18)	0.71409 (12)	0.17991 (12)	0.0186 (3)	
O3	0.48450 (19)	0.53074 (14)	0.34811 (13)	0.0257 (3)	
Cl1	1.27582 (8)	0.11559 (5)	0.51284 (5)	0.03372 (13)	
Br1	−0.46435 (2)	0.79269 (2)	−0.13927 (2)	0.02456 (7)	

### Atomic displacement parameters (Å^2^)

**Table:**

	*U*^11^	*U*^22^	*U*^33^	*U*^12^	*U*^13^	*U*^23^
C1	0.0181 (8)	0.0132 (8)	0.0156 (8)	0.0008 (6)	0.0067 (7)	0.0013 (6)
C2	0.0189 (8)	0.0136 (8)	0.0178 (8)	−0.0008 (6)	0.0069 (7)	−0.0010 (6)
C3	0.0165 (8)	0.0160 (9)	0.0160 (8)	−0.0002 (6)	0.0039 (7)	0.0007 (6)
C4	0.0244 (9)	0.0160 (9)	0.0213 (9)	0.0033 (7)	0.0091 (7)	−0.0003 (7)
C5	0.0288 (9)	0.0157 (9)	0.0196 (9)	0.0016 (7)	0.0078 (8)	−0.0043 (7)
C6	0.0219 (8)	0.0167 (9)	0.0164 (8)	−0.0014 (7)	0.0035 (7)	−0.0012 (6)
C7	0.0166 (8)	0.0178 (9)	0.0159 (8)	−0.0010 (6)	0.0043 (7)	0.0009 (6)
C8	0.0159 (8)	0.0179 (9)	0.0206 (9)	0.0023 (6)	0.0049 (7)	−0.0008 (7)
C9	0.0181 (8)	0.0192 (9)	0.0142 (8)	−0.0007 (6)	0.0035 (7)	−0.0043 (6)
C10	0.0179 (8)	0.0154 (9)	0.0144 (8)	−0.0023 (6)	0.0021 (7)	−0.0035 (6)
C11	0.0217 (8)	0.0187 (9)	0.0204 (9)	0.0004 (7)	0.0071 (7)	−0.0001 (7)
C12	0.0187 (8)	0.0241 (10)	0.0256 (10)	0.0009 (7)	0.0055 (8)	−0.0029 (7)
C13	0.0258 (9)	0.0169 (9)	0.0188 (9)	0.0036 (7)	−0.0041 (7)	−0.0050 (7)
C14	0.0314 (10)	0.0206 (10)	0.0181 (9)	−0.0022 (7)	0.0037 (8)	0.0015 (7)
C15	0.0227 (9)	0.0238 (10)	0.0174 (9)	−0.0021 (7)	0.0058 (7)	−0.0024 (7)
O1	0.0204 (6)	0.0167 (7)	0.0272 (7)	0.0018 (5)	0.0043 (5)	−0.0065 (5)
O2	0.0154 (6)	0.0162 (6)	0.0218 (6)	0.0014 (5)	0.0019 (5)	−0.0017 (5)
O3	0.0224 (6)	0.0325 (8)	0.0262 (7)	0.0046 (5)	0.0127 (6)	0.0037 (6)
Cl1	0.0352 (3)	0.0251 (3)	0.0316 (3)	0.0110 (2)	−0.0049 (2)	−0.0012 (2)
Br1	0.01750 (10)	0.02591 (12)	0.02626 (12)	0.00457 (7)	0.00057 (8)	−0.00652 (8)

### Geometric parameters (Å, º)

**Table:**

C1—C6	1.391 (2)	C8—H8A	0.9900
C1—C2	1.391 (2)	C8—H8B	0.9900
C1—C7	1.488 (2)	C9—O3	1.212 (2)
C2—C3	1.380 (2)	C9—C10	1.490 (2)
C2—H2	0.9500	C10—C11	1.393 (2)
C3—C4	1.377 (2)	C10—C15	1.393 (2)
C3—Br1	1.8995 (16)	C11—C12	1.391 (2)
C4—C5	1.387 (3)	C11—H11	0.9500
C4—H4	0.9500	C12—C13	1.374 (3)
C5—C6	1.386 (2)	C12—H12	0.9500
C5—H5	0.9500	C13—C14	1.387 (3)
C6—H6	0.9500	C13—Cl1	1.7429 (18)
C7—O1	1.202 (2)	C14—C15	1.379 (3)
C7—O2	1.348 (2)	C14—H14	0.9500
C8—O2	1.4283 (19)	C15—H15	0.9500
C8—C9	1.515 (2)		
			
C6—C1—C2	120.59 (15)	C9—C8—H8B	109.7
C6—C1—C7	122.35 (15)	H8A—C8—H8B	108.2
C2—C1—C7	117.04 (15)	O3—C9—C10	121.67 (15)
C3—C2—C1	118.46 (16)	O3—C9—C8	120.25 (15)
C3—C2—H2	120.8	C10—C9—C8	118.08 (14)
C1—C2—H2	120.8	C11—C10—C15	119.46 (16)
C4—C3—C2	122.08 (16)	C11—C10—C9	122.21 (15)
C4—C3—Br1	119.44 (13)	C15—C10—C9	118.33 (15)
C2—C3—Br1	118.49 (13)	C12—C11—C10	120.38 (16)
C3—C4—C5	118.85 (16)	C12—C11—H11	119.8
C3—C4—H4	120.6	C10—C11—H11	119.8
C5—C4—H4	120.6	C13—C12—C11	118.76 (17)
C6—C5—C4	120.58 (17)	C13—C12—H12	120.6
C6—C5—H5	119.7	C11—C12—H12	120.6
C4—C5—H5	119.7	C12—C13—C14	122.01 (17)
C5—C6—C1	119.41 (16)	C12—C13—Cl1	119.05 (15)
C5—C6—H6	120.3	C14—C13—Cl1	118.94 (14)
C1—C6—H6	120.3	C15—C14—C13	118.85 (17)
O1—C7—O2	124.03 (15)	C15—C14—H14	120.6
O1—C7—C1	124.27 (15)	C13—C14—H14	120.6
O2—C7—C1	111.69 (15)	C14—C15—C10	120.54 (17)
O2—C8—C9	109.80 (13)	C14—C15—H15	119.7
O2—C8—H8A	109.7	C10—C15—H15	119.7
C9—C8—H8A	109.7	C7—O2—C8	114.81 (13)
O2—C8—H8B	109.7		
			
C6—C1—C2—C3	0.7 (2)	C8—C9—C10—C11	−0.3 (2)
C7—C1—C2—C3	179.07 (14)	O3—C9—C10—C15	−0.7 (3)
C1—C2—C3—C4	−1.8 (2)	C8—C9—C10—C15	−179.85 (16)
C1—C2—C3—Br1	178.47 (11)	C15—C10—C11—C12	0.5 (3)
C2—C3—C4—C5	1.4 (3)	C9—C10—C11—C12	−179.10 (16)
Br1—C3—C4—C5	−178.85 (12)	C10—C11—C12—C13	0.0 (3)
C3—C4—C5—C6	0.1 (3)	C11—C12—C13—C14	−0.1 (3)
C4—C5—C6—C1	−1.1 (3)	C11—C12—C13—Cl1	180.00 (14)
C2—C1—C6—C5	0.7 (2)	C12—C13—C14—C15	−0.3 (3)
C7—C1—C6—C5	−177.59 (15)	Cl1—C13—C14—C15	179.61 (14)
C6—C1—C7—O1	164.73 (17)	C13—C14—C15—C10	0.8 (3)
C2—C1—C7—O1	−13.6 (2)	C11—C10—C15—C14	−0.9 (3)
C6—C1—C7—O2	−15.9 (2)	C9—C10—C15—C14	178.71 (16)
C2—C1—C7—O2	165.76 (13)	O1—C7—O2—C8	−9.5 (2)
O2—C8—C9—O3	5.4 (2)	C1—C7—O2—C8	171.15 (13)
O2—C8—C9—C10	−175.45 (14)	C9—C8—O2—C7	−75.86 (17)
O3—C9—C10—C11	178.89 (17)		

### Hydrogen-bond geometry (Å, º)

**Table:**

*D*—H···*A*	*D*—H	H···*A*	*D*···*A*	*D*—H···*A*
C4—H4···Br1^i^	0.95	2.97	3.8762 (18)	160
C8—H8*B*···O1^ii^	0.99	2.42	3.396 (2)	168
C15—H15···O3^iii^	0.95	2.60	3.418 (2)	144

Symmetry codes: (i) −*x*−1, −*y*+2, −*z*; (ii) −*x*+1, −*y*+1, −*z*; (iii) −*x*+1, −*y*+1, −*z*+1.
